# Developing “MinDag” – an app to capture symptom variation and illness mechanisms in bipolar disorder

**DOI:** 10.3389/fmedt.2022.910533

**Published:** 2022-07-22

**Authors:** Thomas D. Bjella, Margrethe Collier Høegh, Stine Holmstul Olsen, Sofie R. Aminoff, Elizabeth Barrett, Torill Ueland, Romain Icick, Ole A. Andreassen, Mari Nerhus, Henrik Myhre Ihler, Marthe Hagen, Cecilie Busch-Christensen, Ingrid Melle, Trine Vik Lagerberg

**Affiliations:** ^1^NORMENT, Division of Mental Health and Addiction, Oslo University Hospital and Institute of Clinical Medicine, University of Oslo, Oslo, Norway; ^2^Early Intervention in Psychosis Advisory Unit for South East Norway, Division of Mental Health and Addiction, Oslo University Hospital, Oslo, Norway; ^3^Department of Psychology, University of Oslo, Oslo, Norway; ^4^INSERM, UMR_S1144, Paris University, Paris, France; ^5^FondaMental Foundation, Créteil, France; ^6^Department of Special Psychiatry, Division of Mental Health Services, Akershus University Hospital, Lørenskog, Norway

**Keywords:** eHealth, digital characterization, bipolar disorder, mood, sleep, substance use (drugs, alcohol, smoking), psychotic symptoms, illness course

## Abstract

**Introduction:**

The illness course of bipolar disorder (BD) is highly heterogeneous with substantial variation between individuals with the same BD subtype and within individuals over time. This heterogeneity is not well-delineated and hampers the development of more targeted treatment. Furthermore, although lifestyle-related behaviors are believed to play a role in the illness course, such mechanisms are poorly understood. To address some of these knowledge gaps, we aimed to develop an app for collection of multi-dimensional longitudinal data on BD-relevant symptoms and lifestyle-related behaviors.

**Methods:**

An app named MinDag was developed at the Norwegian Center for Mental Disorders Research in Oslo, Norway. The app was designed to tap into selected areas: mood, sleep, functioning/activities (social, occupational, physical exercise, leisure), substance use, emotional reactivity, and psychotic experiences. Ethical, security and usability issues were highly prioritized throughout the development and for the final app solution. We conducted beta- and pilot testing to eliminate technical problems and enhance usability and acceptability.

**Results:**

The final version of MinDag comprises six modules; three which are presented for the user once daily (the Sleep module in the morning and the Mood and Functoning/Activities modules in the evening) and three which are presented once weekly (Substance Use, Emotional Reactivity, and Psychotic Experiences modules). In general, MinDag was well received in both in the beta-testing and the pilot study, and the participants provided valuable feedback that was taken into account in the final development. MinDag is now in use as part of the research protocol at the NORMENT center and in a specialized treatment unit for BD at Oslo University Hospital in Norway.

**Discussion:**

We believe that MinDag will generate unique longitudinal data well suited for capturing the heterogeneity of BD and clarifying important unresolved issues such as how life-style related behavior may influence BD symptoms. Also, the experiences and knowledge derived from the development of MinDag may contribute to improving the security, acceptability, and benefit of digital tools in mental health.

## Introduction

Bipolar disorder (BD) affects 1–2% of the population worldwide (1, 2). In clinical practice, we observe considerable heterogeneity both between individuals with BD of the same subtype, and within individuals with BD over time. This includes the duration and severity of (hypo) manic and depressive episodes, as well as the duration of euthymic periods. Also, some individuals experience distinct depressive and (hypo) manic episodes with fully euthymic periods in between, while a substantial proportion experience more chronic sub-syndromal mood symptoms and/or mood instability (3, 4). Such mood instability appears to be clinically important as it is associated with a more severe clinical course and disability (5, 6) and may require specific treatment strategies. Still, the current diagnostic system and traditional research methods do not seem well suited to capture this and other phenomena that may be relevant to our understanding of psychopathology. Therefore, a more “bottom-up” approach to characterizing mental disorders has been proposed including fine-grained collection of longitudinal and multi-dimensional clinical data (7). With such data, we may obtain more delineated and clearly defined clinical phenotypes that may also be easier to link with risk factors and biomarkers (8). New digital tools such as smartphone apps and digital wearables have provided novel possibilities for capturing such “bottom-up data,” opening avenues for richer and more ecologically valid characterization (9).

In addition to a more detailed characterization of the variation in BD symptoms over time, there is a need for a better understanding of the mechanisms, i.e., processes and interplays underlying this variation. On top of the contribution of genetic liability to variations in symptoms and illness episodes, individuals' life-style-related behaviors and contextual factors such as life events and social support play a role during the course of BD. For instance, while substance use (10, 11) and sleep disruptions (12) appear to destabilize the course of BD, social regularity/stability, (13) and physical activity (14) appear to stabilize and/or reduce episode severity. However, such putative relationships are to a large extent assumed rather than empirically validated. Thus, a better understanding of the interplay between symptoms and behavior is important to identify efficient targets for intervention. Parallel monitoring of multiple experiences and events has become possible with easily available digital tools such as smartphone apps.

Several apps for mood monitoring in BD have been developed and tested, and current research using these apps has multiple aims. For instance, frequent self-reports have the potential to reveal symptom exacerbations at an early stage and can be powerful clinical tools to detect changes needing prompt intervention. Thus, several research efforts aim to determine the validity of app-based self-reports of mood and/or mood-related behavioral parameters from digital phenotyping (15, 16), and further, whether such data can predict the onset of affective episodes (17, 18). In other studies, app-based self-monitoring of a limited set of mood- and other symptoms is part of a more comprehensive clinical intervention aiming to enhance and personalize patient care, with e.g., targeted psychoeducation or CBT-based feedback (19–23). Currently, the clinical effects of the digitally based interventions are investigated in clinical trials. However, to date, there are few apps that primarily aim to (1) capture the longitudinal variation in mood and other BD-related symptoms within and between individuals with BD and (2) collect data to investigate mechanisms and drivers of such variations. We are only aware of two such app-based studies: Toi-Meme, which is developed to investigate the 3-month interplay between mood and some fundamental dimensions of behavioral activation (motor activity, cognition, and emotional response) (24), and Socialise, with the objective to determine the relationship between circadian rhythm and affective symptoms over a period of 10 weeks (25). Our aim was thus to develop an app that captures a more comprehensive set of symptom- and behavioral dimensions relevant for long-term follow-up of patients with BD for the research purposes described in (1) and (2) above. The selection of parameters for inclusion in the app was based on the aim to develop a tool to monitor the long-term symptomatology of BD as well as the interplay between different symptoms and behaviors. Consequently, we aimed to cover (a) mood and other BD-related symptoms, (b) functioning and (c) lifestyle-related behaviors with potential impact on the illness course. Our aim was to obtain relatively long monitoring periods (6 months) to capture symptom variation as well as the episodic nature of BD (18). Therefore, we focused on balancing the comprehensiveness of the app with acceptability and user-experienced relevance to enhance study retention and to prepare for future clinical use. This development had two phases with the following specific aims: Phase 1; To select and design relevant symptom- and behavior measures and develop a secure and user-friendly app, Phase 2; To conduct a beta-test and a pilot study to investigate the usability of the app and adjust app features based on feedback from users to enhance acceptability, i.e., the MinDag research project.

## Materials and equipment

### Technical development and software

To develop MinDag, the NORMENT project group collaborated with a team of application engineers and visual designers in the Web application group at the University of Oslo's Center for Information Technology (USIT). The framework and content were decided by the project group while engineers and designers determined which features were applicable and conducted the programming. App development was performed in the React Native open-source UI software framework. This allows for deployment across different operation systems, and MinDag was built for both iOS and Android.

### Setup, data security and -management

**Figure 1:**
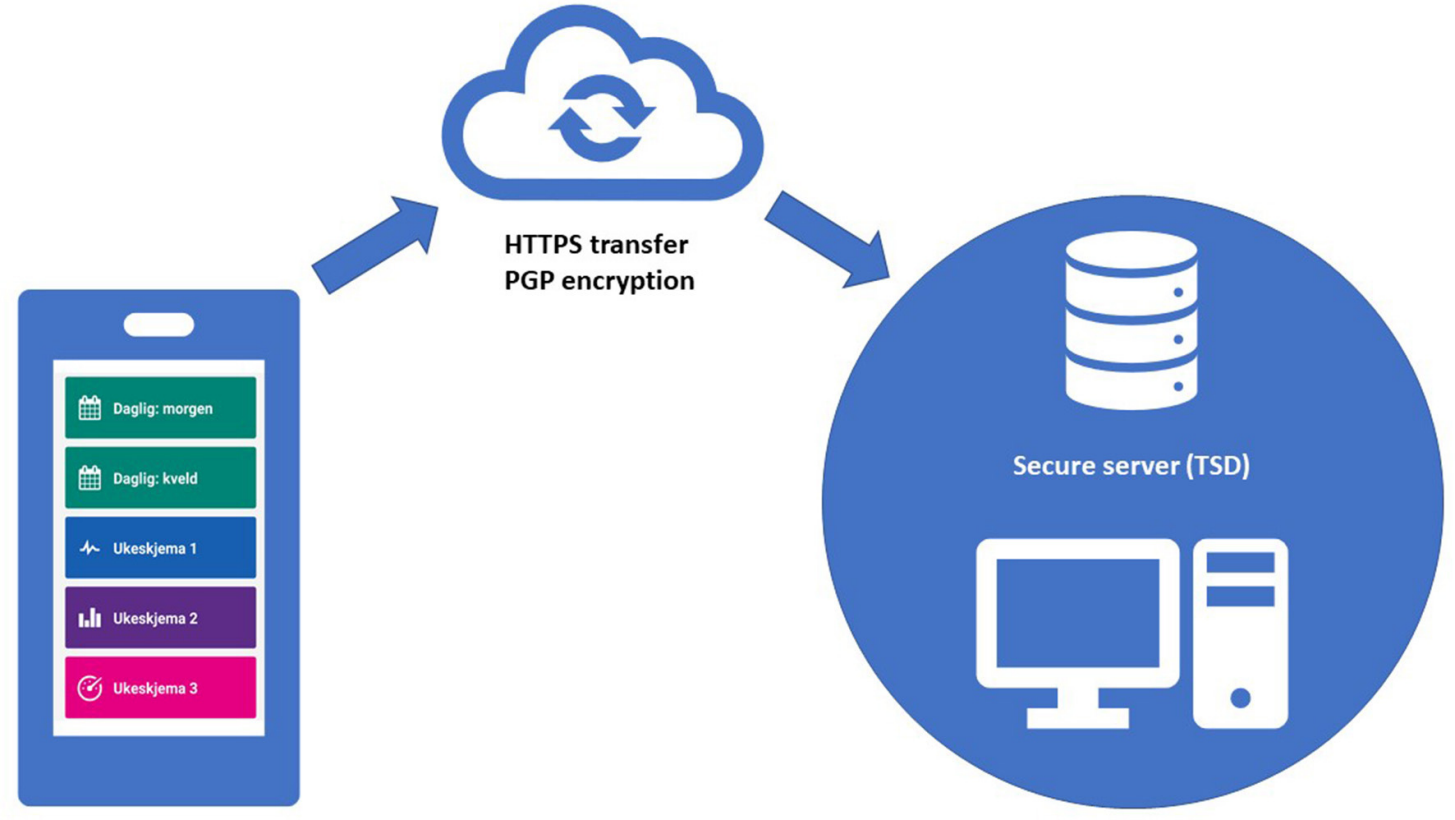
Technical structure for data collection.

Downloading MinDag requires a smartphone with iOS or Android operation system, and a user profile for AppStore or GooglePlay. The system is based on HTTPS transfer of data as JSON files through secure web-survey provider “Nettskjema,” hosted at USIT. Data is not stored on app user devices; for each completed task, data is PGP encrypted and transferred to a secure server (Services for Sensitive Data-TSD), as illustrated in Figure 1. Responses from MinDag are stored as.csv files in a dedicated server on TSD. The generated data will be added to a REDCap offline database and set up for analysis and feedback reports in SPSS v28, R, and RStudio software. Requirements for running analysis tools are recent versions of Windows or Linux OS.

## Methods

### Phase 1

#### Project setting

MinDag was developed at the Center of Excellence Norwegian Centre for Mental Disorders Research (CoE NORMENT) at Oslo University Hospital (OUH) and the University of Oslo (UiO). NORMENT conducts the Thematically Oriented Psychosis (TOP) study, a naturalistic translational study aiming to increase the knowledge about the underlying mechanisms and treatment of severe mental disorders. The study started in 2003 and is still ongoing, with approximately 2000 participants included as of February 2022. The app development is part of the NORMENT's general strategy to expand and improve the study protocol with digital longitudinal data collection. The MinDag development project group consisted of clinical researchers (medical doctors and psychologists) with long-term experience in clinical research on BD, personnel with eHealth technology expertise, and the center's user representative.

#### App content selection

**Figure 2:**
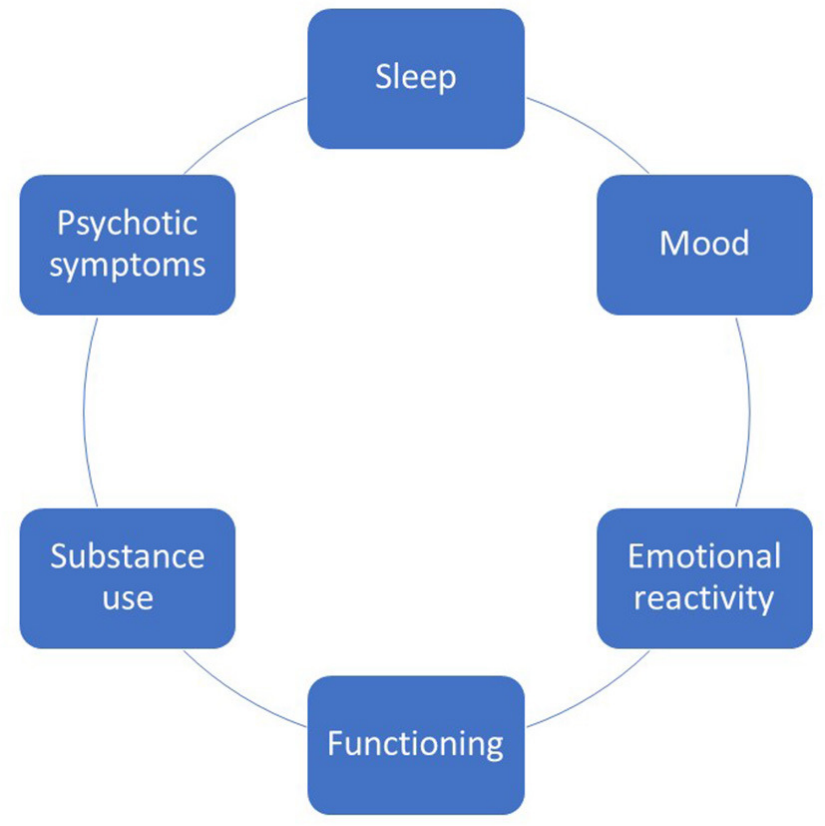
Focus areas selected for inclusion in MinDag.

Six main focus areas, shown in Figure 2, were selected to comprise the modules of MinDag, based on the following rationales:

##### Mood

Monitoring elevated and depressed mood is a *sine qua non* for capturing illness activity in BD. Furthermore, one of the main aims of the app development was to create a tool better suited for capturing mood lability than traditional interviews or questionnaires where individuals are typically asked about their general tendency to shift frequently between different mood states. With such methods, the risk for recall- and generalization bias is large. Mood monitoring *via* an app, on the other hand, can be done continuously i.e., with minimal delay between experience and report in the individual's own environment.

##### Sleep

Disturbances in sleep and sleep/wake cycles are present in both (hypo) manic and depressive episodes (26) and persist between episodes (27). Disturbed sleep in BD is associated with increased relapse rates and reduced quality of life, cognitive functioning, and metabolic health (28–33). The sequence of events is yet unclear: are sleep disturbances caused by a current mood episode, or are they also prodromal to emerging episodes or a causal factor of future relapse (34). Thus, there is a need to better understand the role and mechanisms of sleep disturbances in BD and to develop more valid and feasible methods for assessments. The user-benefits that an app offers in terms of accessibility and immediacy, in addition to the possibility for notifications, may be particularly large when collecting sleep-related information.

##### Psychotic symptoms

A recent meta-analysis reports that psychotic symptoms are present in 58–68% in bipolar I disorder and 14–33% in bipolar II disorder, which may be higher than previously reported (35). Furthermore, the authors conclude there is limited knowledge about the prevalence of psychotic symptoms in bipolar II disorder and in bipolar depression. While the typical psychotic symptoms of mania such as grandiose delusions but also mood-incongruent delusions, thought disorder, hallucinations and catatonia are relatively well characterized (36), less is known about the most common types of psychotic symptoms in mixed and depressive episodes. Also, to what extent psychotic symptoms in BD are driven by behavioral factors such as sleep disturbances, mood lability and substance use is not well known. Thus, more insight into the prevalence, types, fluctuations, and drivers of psychotic symptoms in BD is needed.

##### Emotional reactivity

Emotional reactivity refers to the threshold for, and magnitude of, emotional responses to salient environmental stimuli and can be classified on a dimension ranging from emotional hyper- to hypo-reactivity (37). Hypo-reactivity (inhibition) is common in depression, whereas (hypo) mania and mixed episodes are characterized by hyper-reactivity (activation) (38). However, hyper-reactivity may be present also in depression and is then associated with negative outcomes such as suicidality and substance use (39). Different reactivity clusters have further been identified in patients with subthreshold mood symptoms, and abnormal reactivity levels appear to be associated with higher levels of high-sensitivity C-reactive protein, cardiovascular disease risk as well as functional and cognitive impairments (40, 41). The longitudinal course of emotional reactivity has, however, not been investigated. Unresolved questions include whether changes in reactivity is a prodrome of episode recurrence, if individuals have trait-like tendencies for hyper- and hypo-activation and whether such putative traits influence the BD course including risk for relapse.

##### Substance use/craving

Substance use is common in individuals with BD and comorbid substance use disorders are present in up to 40% of this patient group (42, 43). Comorbid substance use disorders and other maladaptive patterns of substance use including the use of tobacco, appear to destabilize the course of BD and yield more severe outcomes. Such comorbidities have been associated with more frequent illness episodes and poorer functioning (10, 11), increased affective lability (44), increased suicide rates (45, 46), and reduced life expectancy (47). Taken together, it seems that (i) substances may be initiated and used as an attempt to alleviate symptoms of BD, while (ii) the long-term consequences are increased symptom severity and risk of transition from substance use to substance use disorder, and (iii) there is a large inter-individual variability in the risk of adverse consequences associated with substance use in BD. It is noteworthy that some of these relationships still lack empirical evidence despite being commonly observed in clinical practice. As the continued use of substances by individuals with BD may seem paradoxical, a better understanding of the initial mechanisms for substance use and substance use disorder in BD is needed.

##### Functioning/activity

BD is one of the leading causes of disability worldwide (48) and functional impairment is considered to be a common feature of the disorder (49, 50). There is however a large degree of individual variation reflected in a wide range of functional outcomes. Some individuals show superior functioning while others experience significant difficulties in a variety of daily activities, including employment, education and social functioning (49–51). Illness-related factors that have been shown to be associated with functional outcomes include affective symptoms (52), sleep disturbances (53) and substance misuse (54). Less is known about the temporal relationship between these factors and functioning in BD. Functioning in terms of participation in occupational-, social-, leisure- and physical exercise-related activities may also have beneficial effects on the BD symptoms and course. Increased knowledge about the complex interplays between symptoms and functioning/activities may help carve the way for interventions that enhance both functional and symptomatic recovery in BD.

#### App naming and motivational features

The app was given the name “MinDag,” which means “my day” in Norwegian, to signal that the intention of the app was to capture daily personal experiences and routines while also being neutral i.e., not implying psychopathology. We also decided to develop simple gamification features to increase the users' motivation to complete the intended registrations.

### Phase 2

#### Beta-testing

The first beta-testing of MinDag was conducted with a group of four users with lived experience of severe mental disorders, recruited through the NORMENT User Council. At the time of the beta-testing, four of the six MinDag modules had been developed: Mood, Sleep, Emotional reactivity, and Psychotic symptoms. The purpose of the beta-testing was to test the technical functioning and receive feedback about the user experience of MinDag, and next, evaluate and further develop the app. After 4 weeks of testing, the test users were asked for feedback using a semi-structured telephone interview. Themes covered in the interview were technical issues, perceived adequacy of the number of questions and notifications, time spent completing the registrations, and perceived relevance of the content and questions in the different modules. The test users were also asked for any additional feedback. The results of the beta-testing were discussed in a meeting with the technical developers and two of the beta-testing participants.

#### Pilot study

After the initial beta-testing phase, a pilot study was launched. Here, the aim was to test what we considered to be the final version of the app. We were particularly interested in uncovering usability issues, paying attention to user experiences, content relevance and general acceptability. In addition to the participants of the beta-testing, new participants were recruited from the Norwegian Bipolar Association through an invitation in their monthly newsletter. Other people with BD who had previously expressed an interest in the project were also invited. In total, nine participants were recruited. An initial meeting was held where the participants downloaded the app and were given information about the project. The participants then used MinDag for 2 weeks, after which they received a phone call from one of the researchers. They were asked about their overall experience with the app and to report any technical problems. After another 2 weeks, all the participants were invited to a focus group meeting. Five participants attended this meeting, where feedback was collected through individual post-it notes, addressing pros and cons for each module, followed by a group discussion and a questionnaire. The questionnaire consisted of the same questions as those used in the beta-testing, with a few additions, such as whether using MinDag had affected their mental health in a negative way, if information about the previous nights' sleep was difficult to remember in the evening, and what they thought about the gamification elements.

In addition to user involvement in the beta- and pilot testing, the MinDag development has been discussed with members of the board of the Norwegian Bipolar Association who has expressed a need for quality-controlled apps targeted at individuals with BD. Also, a total of four user representatives employed at NORMENT, two of whom were specifically recruited to join the MinDag project group, have been involved in different phases of the development. Lastly, the project group has performed continuous evaluation and testing during all phases of the development.

## Results

### Phase 1

#### MinDag content

To cover each of the selected dimensions, we searched for validated self-report measures which were adapted to an app format or could potentially be digitized to cover the selected parameters. If such measures were not available, we developed suitable assessments. Here, we describe the final content and functions of the MinDag, i.e., after changes were made based on beta- and pilot testing. Please refer to Table 1 for an overview of the MinDag content.

**Table 1:** Description of the different app-modules, and the theoretical, empirical, and clinical basis.

**Module/Frequency**	**Target measure**	**Items**	**Scale**	**Basis of measure/scale used**
Sleep/Daily	Sleep duration and - quality	Time went to bed Time fell asleep Time woke up in the morning Hours awake during night Hours sleep during daytime Quality of sleep Did you feel rested in the morning	hh.mm. Number of hours (in half hours) Time-range, minutes/hours intervals Likert scale 1–7 (“Very poorly”–“Very well”) Yes/no	Developed by project group
Mood/ Daily	Experienced levels of different mood types	Sadness Irritability Happiness Anxiety Panic Mood elevation Anger	Likert scale 1–7 (“Not at all” – “To a large degree”) “ “ “	MAThyS
Function and Activities/ Daily	Time spent on different types of everyday activities	Work/studies Rehabilitation/work training Hobbies/leisure activities Social interaction Digital social interaction Physical exercise Contention with daily activity	Time-range (hour intervals) “ “ Time-range (15 min-h intervals) “ “ Likert scale 1–7 (“To a small degree”–“To a large degree”)	Developed by project group
Substance use/ Weekly	Consumption and craving of substances	Use of alcohol, nicotine, illicit drugs Days of alcohol use, units Units of nicotine products Days of use of selected drugs, doses Degree of craving for alcohol, nicotine or illicit drugs if not used	Yes/no (for each substance, incl. 8 types of illicit drugs) No. of days used, total no. of units Average no. of units per day No. of days used (for each drug), total no. of doses Craving yes/no, if yes: Likert scale 1–7 (“Little”-Extreme”)	Developed by project group
Psychotic symptoms/ Weekly	Delusions and hallucinations, presence, levels and functional impact	Auditory hallucinations Visual hallucinations Grandiosity Suspiciousness Selected delusion 1 Selected delusion 2	Presence: Yes/no. If yes: Three statements about functional loss scored on Likert scale 0–6 (“Not at all” – “To a large degree”) Three statements scored on Likert scale 1–7 For suspiciousness item and selected delusion 1 and 2: Degree of presence scored on Likert scale 1-7 (“Not at all” – “To a large degree”). If 3 or above: Three statements about functional loss scored on Likert scale 1–7 (“Not at all”–“To a large degree”)	ClinTouch
Emotional reactivity/ Weekly	Emotional responses to environmental stimuli	20 items with two opposite statements	Visual analog scale from 1–10 representing a continuum from hypo- to hyper-reactivity)	MAThyS

##### Mood

**Figure 3:**
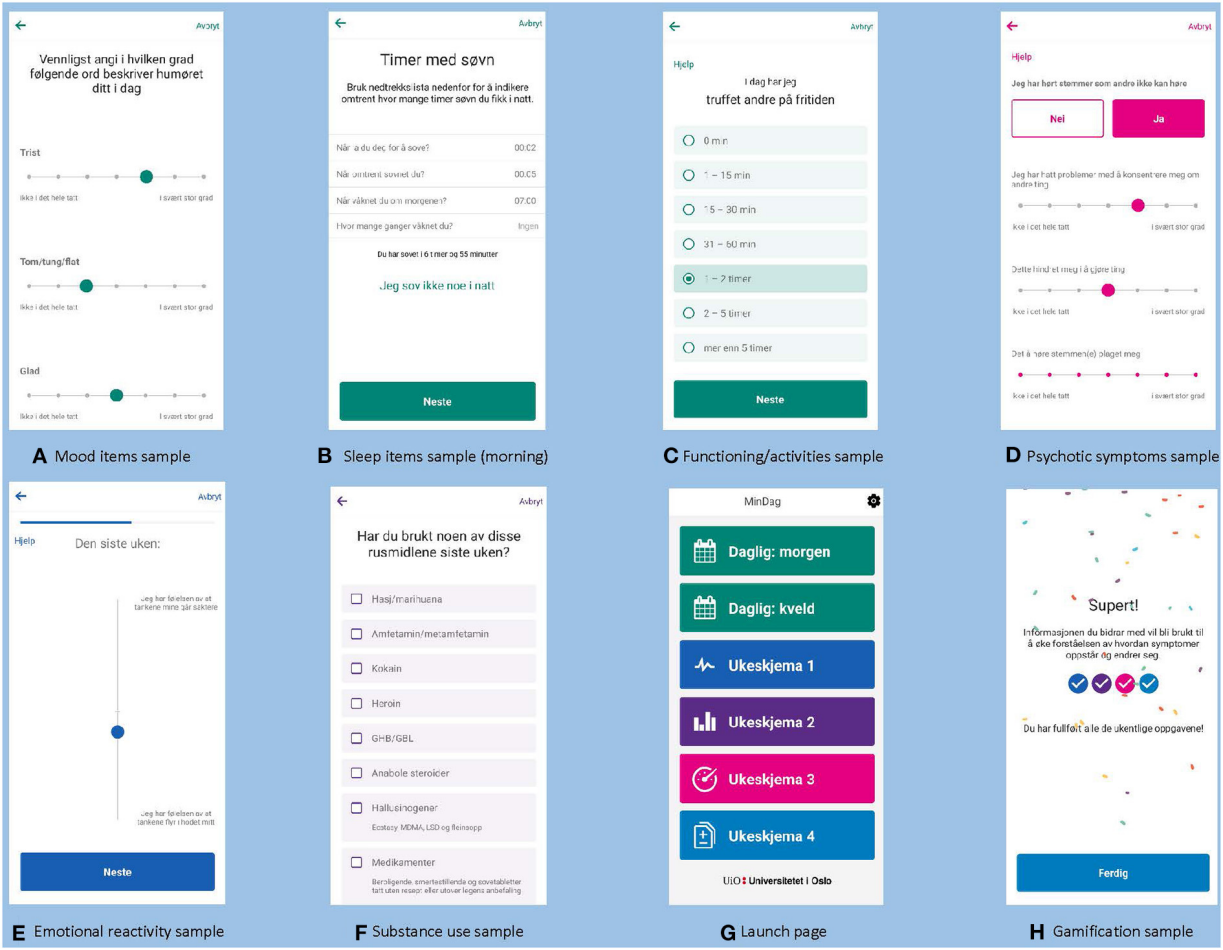
Screenshots from MinDag modules.

As we had a specific focus on exploring symptom variation including mood instability, simple single- or double items for evaluating mood was chosen as opposed to comprehensive assessments of syndromal conditions of depression and (hypo) mania. For the same reason, we followed recommendations to monitor mood on a daily (as opposed to weekly) basis (16, 55). For the specific mood monitoring items, we decided to use a sub-scale of the Multidimensional Assessment of Thymic States Scale (MAThyS) (56) with 7 items assessing the degree of happiness, sadness, anger, irritability, mood elevation, anxiety and panic on a 7-point Likert scale from “not at all” to “to a very large degree” (Figure 3A). The mood items are presented in the evening. Previous studies have demonstrated satisfactory correspondence between self-reported mood and clinician-based evaluations with validated scales for depression and mania. However, to investigate the validity of the mood ratings used in MinDag, we planned for clinician ratings of depressive and (hypo) manic symptoms at baseline and 3-month follow-up with the Quick Inventory of Depressive Symptomatology–Clinician Rated (57) and the Bech-Rafaelsen Mania Scale (58), respectively.

##### Sleep

The sleep module was developed by the project group but in correspondence with the Consensus Sleep Diary, a validated method to collect subjective measures of sleep and diagnose some sleep disorders (59, 60). The items cover the time of going to bed, sleep on- and offset, number and total duration of wake-ups during the night, the experience of feeling rested, and subjective quality of sleep (Figure 3B). Together, these items provide data that enables estimation of common sleep parameters, such as sleep onset latency, total sleep time, wake after sleep onset (61). The accuracy of sleep diaries has proven to be satisfactory (62). Questions about sleep during the night are prompted each morning while questions about day-time sleep are presented in the evening.

##### Functioning/activities

This module was developed by the project group. Items were developed to tap areas of everyday functioning often found to be impaired in severe mental illness while also covering activities with a possible beneficial impact on the BD course. Items capture time spent on school/work, work training, rehabilitation and/or treatment, physical exercise, hobbies/leisure activities, and time spent with other people either physically or on the phone/online (Figure 3C). The module also contains a question addressing one's satisfaction with the day's activities. The questions are presented daily in the evening.

##### Psychotic symptoms

To monitor psychotic symptoms, we used selected items from the ClinTouch system (63) which is based on the Positive and Negative Syndrome Scale for Schizophrenia (64). This use was approved by the ClinTouch developers. The items were translated into Norwegian using standard methods for translation (i.e., approval of back-translation by the developers). Items were selected to cover positive symptoms i.e., delusions and hallucinations (auditory and visual) (Figure 3D). Questions about the presence (yes/no) of hallucinations are presented by default each week, and if the participant confirms such experiences, follow-up items to evaluate to which extent the symptom affects functioning or causes distress will emerge. Questions about grandiosity and suspiciousness are presented by default each week, while other delusional experiences are initially selected for monitoring only if they have ever been experienced by the participant. A list of 12 different delusion themes is presented for selection, and a maximum of two delusions can be selected, based on a discussion between the participant and the clinical researcher with detailed knowledge about the participant's illness history (including psychotic symptoms). For participants who have experienced three or more delusions, the two delusions associated with the greatest conviction and distress are selected. If a delusion is rated 3 or more on a Likert scale from 1 (“not at all”) to 7 (“to a very large degree”), follow-up items to evaluate to which extent the symptom affects functioning or causes distress will emerge. This module is presented once weekly.

##### Emotional reactivity

To monitor emotional reactivity, we used the MAThyS (56) which has been found to have good psychometric properties (38, 65). The use in MinDag was approved by the developers of MAThyS. The scale was translated into Norwegian using standard methods for translation (i.e., approval of back-translation by the developers). MAThyS is a visual analog scale consisting of 20 items covering five dimensions typically affected in mood episodes: cognition, motivation, sensory perception, emotional reactivity and psychomotor activation/retardation. The dimensions can fluctuate from inhibition to excitation, and participants are asked to mark their states for the preceding week for all items on a line between two predefined extreme propositions (Figure 3E). The scale yields a total score of inhibition/activation and five subscores, one of which specifically pertains to level of emotional reactivity. In addition to the MAThyS, the module also contains a question of whether something happened during the last week that the user specifically feels affected by, with an open text field where a short description can be reported. MinDag prompts the participant to complete this module on a weekly basis.

##### Substance use and craving

This module was developed by the project group and includes weekly assessments of use of alcohol, nicotine products, and the most commonly used illicit substances (incl. medication with addictive potential used differently than prescribed), number of days of use, number of units/doses consumed in average, and degree of craving (Figure 3F). Although there is a risk for recall bias with weekly (as opposed to daily) monitoring, we chose this frequency to keep the load on the participant at an acceptable level. Moreover, we anticipated that most participants would not use substances on a daily basis. We also considered that counting the number of units/doses consumed during the last week to be feasible for most participants. Self-report of substance use has been shown to correspond well with biological measures (66).

#### General design and motivational features

Participants must allow MinDag app-notifications on their smartphone to be prompted for scheduled tasks. “Help”-buttons within the modules were developed with instructive texts to enhance reliability. All daily items in the app were set to be open for response until the next day's questions were opened, i.e., for 24 h. All weekly questions were finally set to be open for response for 36 h (extended from 24 h in the initial set-up). The final version of the launch page with all modules are presented in Figure 3G. In the current app set-up, all modules are to be completed by all participants. In the early developmental phase, we considered providing feedback within the app in terms of visualizations of e.g., sleep hours and mood ratings. However, there were technical barriers for developing such features at the time of the development. Instead, we developed an algorithm for exporting visualizations/curves based on the data which could be presented for the participants at follow-up appointments. We also developed gamification features where “streaks” can be obtained for completed modules, and for completing a week of daily submissions. After completion, the screen lights up with colorful confetti. In addition, motivational texts were presented in the streak overview where messages such as “Super! The information you provide will be used to increase our understanding about how symptoms evolve and change.” and “Thank you! Your contribution is very valuable and will help us improve the quality of prevention and treatment in the future.” (Figure 3H).

#### Ethics and data privacy

Participants download MinDag to their private smartphones. To unlock the app, the users' personal passcode or face-recognition is needed. Since notifications are easily visible on the screen, they are neutrally formulated with the message “You have a new task to complete.” The TSD data transfer and storage solution for MinDag and the app content have been approved in risk- and vulnerability evaluation by the Data Protection Officer at Oslo University Hospital. There is a safety-feature in the app that holds the completed module-submission for transfer until a network is available. The HTTPS transfer is stable and invulnerable with PGP-encryption of the data. In the beta- and pilot testing, potentially sensitive data collected *via* the app was anonymous as participants created random study-IDs, undisclosed to the research personnel. For the formal MinDag study, we developed an alert algorithm within the TSD server to detect if a participant reports high and enduring symptom levels. The alert-dashboard will be reviewed weekly by study nurses in the project. In case of alerts, a clinical researcher (doctor or psychologist) will contact the participant to check whether they have an appointment with their treating clinician in the near future or if any measures need to be taken to ensure necessary care. The study has been approved by the Regional Ethics Committee (REK 2018/208).

### Phase 2

#### Beta-testing

Feedback from the beta-testing revealed technical issues that prevented two of the users from completing MinDag registrations on a daily basis. In addition, several problems with the notifications were reported. Apart from these issues, the beta-testers found the app easy to use and understand, and the questions were considered relevant. Furthermore, the testers considered time spent engaging with the app as acceptable, and they responded positively to suggestions of adding a few more questions. They proposed to add daily questions about diet, physical activity and some more details about sleep duration and -quality. All the testers expressed a desire to get feedback about their responses within MinDag but were also positive to the possibility of personal feedback through regular meetings with research clinicians. During the beta-testing interview, the test-users were informed about the plan for adding a module on substance use, to which they responded positively. After the beta-testing phase, the technical problems were resolved and the following additions were made to the app: A question about whether one had not slept at all during the night, the Substance use module and the Functioning/activities module.

#### Pilot study

##### Technical errors/usability issues

In general, participants reported that they found MinDag easy to use. Some participants reported receiving more notifications than intended, while others reported receiving fewer. Other minor technical issues were also uncovered. The participants suggested to increase the time limit for response to the weekly questions, extending the response time to 36 or 48 h. In the Emotional reactivity module, the participants found it difficult to determine the middle point on the sliding scale and requested this to be more clearly indicated. Some participants found the text in this module too small to read. It was also suggested to add more colors to this module. Some participants reported trouble remembering their sleep behavior from the previous night. On the other hand, the participants generally reported that they found it easy to remember their week when answering questions in the weekly modules. Finally, the gamification elements were well received, and some participants suggested adding even more such features.

##### App content/user experience

Participants reported some individual differences in perceived relevance of the questions, particularly pertaining to the questions regarding psychotic experiences. Some participants also found the daily questions somewhat repetitive. A question was raised regarding the meaning of the Norwegian word for “elevated,” where the intention was to capture (hypo) manic mood states. The participants also suggested adding questions about stress, if life is worth living, suicidality and whether something out of the ordinary happened recently. Additionally, the possibility to add questions with personal relevance was suggested to increase the motivation to use MinDag. Responses concerning the number of daily questions varied between “too many” and “two to five questions may be added.” Regarding the number of weekly questions, the responses were “ok” and “five to ten questions may be added.” Participants did not report that using MinDag affected their mental health negatively, although one participant described sometimes feeling a bit surveilled, and another felt irritated. The participants were generally positive about the prospect of using the app for 6 months.

##### Revision of MinDag based on pilot feedback

Based on the focus group meeting, the following changes were made to the app: The daily questions were split into two, resulting in a morning module and an evening module where items about sleep the night before were placed in the morning module. We also added two more items in the sleep module, and the interval of sleep at daytime was refined. Two words were added to the original single word for mood elevation to clarify the experience we aimed to capture. A question about the degree of satisfaction with today's activities was added in the Functioning/activities module. A question with an open response text field was added at the end of the Emotional reactivity module with the possibility to describe particular experiences that has affected the user the last week. Furthermore, some technical features were revised to increase usability, such as more space between items for easier scrolling, larger text in some places and markers for middle points. The possible response time for the weekly modules was extended from 24 to 36 h. Questions measuring stress, as suggested by the participants, were not added as we considered that this would require an additional module that would add too much to the total MinDag response time. Questions about suicidality were not added as we deemed that this would require a closer follow-up of the data and participants than what was feasible with the available resources of the project. After these changes, we concluded that MinDag had satisfactory content and acceptability, and was ready to be launched.

#### Current status and future plans

MinDag is currently available in Norwegian language only and restricted to participants recruited to a NORMENT-based study. Formal data collection with MinDag started in September 2019, mainly in a specialized clinical unit for BD at Oslo University Hospital but also in general secondary psychiatric services in the Oslo area. Inclusion criteria are a DSM 5 diagnosis of a bipolar spectrum disorder and age between 18 – 65 years. Exclusion criteria are brain damage (head injury with hospitalization) and lack of knowledge of a Scandinavian language. Due to the COVID-19 pandemic (initial lock-down of services, infection control procedures etc.), recruitment rates to date have been relatively slow with 37 participants included as of February 2022. Of these, 23 have completed their use of MinDag by 1^st^ June 2022, and demographic and clinical characteristics as well as basic data on use periods and adherence are presented in Table 2. We expect that recruitment will speed up from spring 2022 and aim for approximately 100 participants by the end of 2024. The attrition rate is estimated to 15%, i.e., we expect approximately 85 participants to complete the study. User experiences and input is still collected from all participants with the System Usability Scale (67) and a self-constructed feedback survey at the end of the 6-month data collection period in order to prepare for further improvements as well as adaptations for clinical use.

**Table 2:** Characteristics and use data for the first 23 participants included in the MinDag study.

Sex, female, n (%)	14 (61)
Age, mean (SD)	32.5 (9.1)
Bipolar disorder type, *n* (%)	
Bipolar I disorder Bipolar II disorder Bipolar disorder NOS	8 (34.8) 13 (56.5) 2 (8.7)
Total period of app use in calender days, mean (SD) Min – max use period (calender days)	156.8 (62.0) 59–316
Proportion of days of app use within use period, mean (SD)	0.72 (0.15)
Use period, n (%), min – max use period (calender days)	
Less than 6 months ≈ 6 months More than 6 months	12 (52.2) (59–161) 6 (26.1) (176–191) 5 (21.7) (201–316)

*SD, standard deviation; NOS, not otherwise specified*.

##### Preliminary data on user characteristics, use length and adherence

As seen in Table 2, about half of the participants ended their use period before 6 months while the other half used the app for 6 months or considerably longer. The adherence rate of the daily module was 72%. There was no significant correlation between adherence rates and the length of the use period (i.e., proportion of days with complete data during the use period); Spearman's rho 0.013, *p* =0.95.

#### Data analysis

The app-based data generates large quantities of data for each participant, allowing testing of associations in a hypothesis-driven as well as data-driven analyses. In line with a few recent studies on mood instability in BD, we will apply the root mean square successive difference (rMSSD) method to capture both the magnitude and the temporal order of changes in mood (18, 68). We will also calculate “mood instability factors”: the number of mood changes divided by the number of weeks followed for the main mood states of BD, namely depression, elevation, and irritability (69). As missing data is a challenge with frequent longitudinal self-reports, this is an additional motivation for the monitoring of submissions during the study-period. However, when monitoring symptoms on a daily basis, missingness is to some degree expected and will not have major implications for all types of research questions. When missingness is a problem, it will be treated with suitable imputation methods. Data may be assumed to be missing not-at-random if mood levels are high the last days leading up to a period of missing data. The availability of longitudinal data series with resolutions ranging from days to weeks may also enable the implementation of causal inference models. For this purpose, we will have the option of introducing the time dimension both implicitly, e.g., guiding the design of appropriate structural equation models, and explicitly, e.g., using vector autoregression or counterfactual modeling, such as machine learning frameworks.

## Discussion

In this paper, we aimed to describe the development of MinDag, an app for long-term monitoring of symptoms and lifestyle-related behaviors in BD. Specifically, we present the overall purpose of the app development, the rationale behind the choice of modules included, the developmental process including beta- and pilot testing and the final product. Important ethical considerations and security solutions are also described. To our knowledge, this is one of the first apps developed to date for parallel monitoring of a wide range of symptoms and behavior in BD. The data generated from MinDag will provide unique opportunities to investigate how mood, sleep and other symptoms fluctuate over time. Furthermore, the data will enable exploration of the interplay between symptoms and lifestyle behaviors such as substance use and participation in occupational, social and physical activities. As MinDag is used as part of the study protocol at NORMENT, the data may also be used in combination with data from other types of assessments: e.g., exposure to early environmental risk factors, neurocognitive function and genotypes and other biomarkers. We expect to contribute to an increased understanding of different mechanisms underlying symptom variation over the course of illness.

The aim of the MinDag development was to produce a tool for comprehensive data collection, i.e., not a clinical tool as part of a treatment intervention. However, we considered the involvement of users with lived experience of BD as crucial ensuring that the app would be well received by the target group. This is necessary to obtain as complete data as possible and to enable long-term use. Preliminary user data show that the length of participation varies with some participants using the app for longer and some for shorter than the 6 months they are initially asked to use it. We consider that periods shorter than 6 months will also yield valuable data and that some variation in use lengths is not a major concern. Thus, encouraging 6 months of use while also being flexible regarding use length appears as an adequate strategy moving on. An adherence rate of 72% is acceptable although somewhat lower than expected compared to a previous similar study (16), and may be partly related to technical difficulties which now have been resolved. This illustrates the importance of monitoring user data and providing feedback and encouragement to the participants throughout the study period.

Also, as MinDag provides highly clinically relevant output, we had future clinical use in mind during the development. Overall, MinDag was well-received by the test users. They engaged actively in both the beta-testing and pilot study, and we were able to revise MinDag according to most of the feedback they provided. Thus, we believe to have found a good balance between scientific ambitions and acceptability, where the latter is particularly important for obtaining data of sufficient completeness and reliability. In this patient-group we can expect variability in motivation and insight that may affect the level of engagement with the app and reliability of data. For instance, symptom peaks in both affective polarities may reduce the likelihood of using the app. Being aware of this, the MinDag team will monitor submissions weekly. The monitor dashboard is also rigged to alert the team after 3 days of missing data, and participants will be contacted by phone and asked about the reason for non-adherence.

Throughout the development period, there has been a great deal of activity in the field of eHealth for mental disorders including some specific developments for BD. Several challenges have been identified with available apps (70), and some are still to be resolved, including lack of standardized ways of reporting data from app-based studies (71). In the current development, we have invested considerable resources into developing measures that are likely to yield valid and high-quality data. Although some previous studies have demonstrated a good correspondence between app-based self-report of mood symptoms and clinician-based evaluations in BD (72), more evidence is needed as validity may differ across settings and the specific measures used. Thus, we have planned for evaluating the validity of the mood measures used in MinDag as part of the formal study. Furthermore, the development has been conducted in a multi-disciplinary team of clinicians, technicians, and user representatives with lived experience. We believe this is key in the development of new digital tools for mental health as different perspectives must be taken into consideration while also taking advantage of the potential of smartphone technology. Apps carry other possibilities of capturing illness activity through in addition to convenient self-report assessments, such as passive monitoring of phone user-behavior. So far, we have chosen not to develop measures for passive data collection as part of MinDag, a decision primarily based on data security and ethical considerations. Such data are transferred to the servers of the phone companies, with limited control of privacy issues in the hands of the researchers. Also, BD is a disorder where the likelihood of consenting to collecting personal data passively will vary over time.

In MinDag, we have not developed feedback features in terms of visual illustrations of the user's registrations for two reasons: Firstly, the main purpose of MinDag is to obtain naturalistic data, thus we were reluctant to include features that may serve as an intervention. Providing feedback about the interplay between symptoms and behavior may have psychoeducational effects, although recent studies indicate that such an effect is limited, at least on primary outcomes such as affective recurrence and hospitalizations (19, 73). Secondly, there have been indications that app-based follow-up with integrated feedback is associated with a higher risk for depressive episodes, which may be caused by a reinforcing effect of continuous reminders about one's own depressive state (19). Consequently, we decided to present visual illustrations based on individual data at follow-up visits with research clinicians for participants who were interested.

Although most of our participants did not report that use of the app had any negative effects on their mental health, one participant described feeling somewhat surveilled and another feeling irritated. Possible “side-effects” of monitoring personal experiences with an app should be taken seriously. However, we believe that such side-effects can be reduced or even eliminated through measures such as explanations of security issues and personal adaptations of app content (e.g., de-activation of modules). Thus, dialogue with the participants during the study period is vital. This is important also to encourage participants to continue engagement with the app even during periods with high symptom intensity.

We are currently adapting MinDag for use in other patient populations, such as people with schizophrenia. Thus, additional modules are under development, and the MinDag set-up will be flexible in the sense that different modules can be activated/de-activated. MinDag is also currently being included in the protocol of a clinical trial, where it enables continuous monitoring of symptomatic change. This may serve as a valuable supplement to traditional (less frequent) paper-and-pencil-based evaluations.

Technical problems have emerged in all phases of the current project. When users experience bugs and other technical difficulties, the motivation to use the tool declines rapidly. Hence, access to the necessary technical competence and resources is crucial not only in the developmental phase but also for maintenance.

In conclusion, we believe that the unique data that we will obtain from the current project, together with the experiences and knowledge derived from its development, will contribute to improving the benefit, acceptability, and security of digital tools in mental health.

## Data availability statement

The original contributions presented in the study are included in the article/Supplementary material, further inquiries can be directed to the corresponding author.

## Ethics statement

The studies involving human participants were reviewed and approved by Regional Comittee for Medical Research Ethics South East Norway (REK 2018/208). The patients/participants provided their written informed consent to participate in this study.

## Author contributions

IM and TL initiated the project with support from OA. TL and IM outlined the MinDag rationale. SA, MC, EB, MH, TB, SH, HM, MN, CB-C, and TL constituted the project working group. SA, MC, TB, MH, and TL organized the beta-testing and pilot study. TB and TL supervised the technical development. The first manuscript draft was co-written by all members of the project working group plus RI, refined by TB and TL and revised and approved by all co-authors. All authors contributed to the article and approved the submitted version.

## Funding

The project was financially supported by Oslo University Hospital, the University of Oslo, the Research Council of Norway (#223273 and #288542), and the South-East Health Authority (#2000189-19). The funding organizations had no role in the design of the study, the collection, analysis and interpretation of the data, the writing of the report, or in the decision to submit the paper for publication.

## Conflict of interest

OA has received speaker's honorarium from Lundbeck and Sunovion and is a consultant to HealthLytix. RI has been consultant for Indivior, Lundbeck (<2017), talk for Pierre Fabre and in 2019 with fees paid to the CSAPA “Espace Murger” association, regular trainer at the IREMA institute.

The remaining authors declare that the research was conducted in the absence of any commercial or financial relationships that could be construed as a potential conflict of interest.

## Publisher's note

All claims expressed in this article are solely those of the authors and do not necessarily represent those of their affiliated organizations, or those of the publisher, the editors and the reviewers. Any product that may be evaluated in this article, or claim that may be made by its manufacturer, is not guaranteed or endorsed by the publisher.
