# Supplementary material for: Developing “MinDag” – an app to capture symptom variation and illness mechanisms in bipolar disorder
Source: Front Med Technol. 2022 Jul 22;4:910533. doi: 10.3389/fmedt.2022.910533 (PMC9354925; doi:10.3389/fmedt.2022.910533)
Supplement: Supplementary file 1 [file Table_1.docx]

Supplementary material

Code and technical information about the development of MinDag available at

https://www.uio.no/english/services/it/research/data-collection-and-analysis/mobile-applications/index.html
